# People Favour Imperfect Catching by Assuming a Stable World

**DOI:** 10.1371/journal.pone.0035705

**Published:** 2012-04-25

**Authors:** Joan López-Moliner, Matthias S. Keil

**Affiliations:** 1 Departament de Psicologia Bàsica, Universitat de Barcelona, Catalonia, Spain; 2 Institute for Brain, Cognition and Behavior (IR3C), Catalonia, Spain; Bielefeld University, Germany

## Abstract

The visual angle that is projected by an object (e.g. a ball) on the retina depends on the object's size and distance. Without further information, however, the visual angle is ambiguous with respect to size and distance, because equal visual angles can be obtained from a big ball at a longer distance and a smaller one at a correspondingly shorter distance. Failure to recover the true 3D structure of the object (e.g. a ball's physical size) causing the ambiguous retinal image can lead to a timing error when catching the ball. Two opposing views are currently prevailing on how people resolve this ambiguity when estimating time to contact. One explanation challenges any inference about what causes the retinal image (i.e. the necessity to recover this 3D structure), and instead favors a direct analysis of optic flow. In contrast, the second view suggests that action timing could be rather based on obtaining an estimate of the 3D structure of the scene. With the latter, systematic errors will be predicted if our inference of the 3D structure fails to reveal the underlying cause of the retinal image. Here we show that hand closure in catching virtual balls is triggered by visual angle, using an assumption of a constant ball size. As a consequence of this assumption, hand closure starts when the ball is at similar distance across trials. From that distance on, the remaining arrival time, therefore, depends on ball's speed. In order to time the catch successfully, closing time was coupled with ball's speed during the motor phase. This strategy led to an increased precision in catching but at the cost of committing systematic errors.

## Introduction

**Figure 1:**
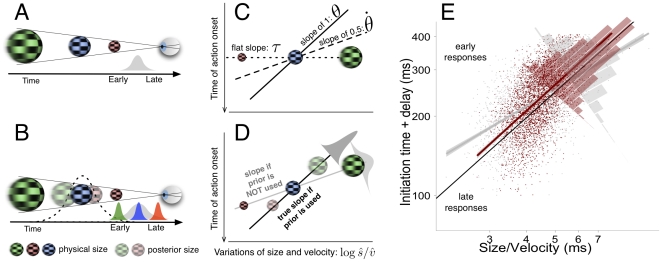
Ambiguity of optical information and predictions. (A) Inherent ambiguity of monocular optic flow. Three balls (non-transparent) of different sizes (colour for illustrative purposes only) and velocities (blur coded) having the same size to velocity ratio (*s*/*v*) will arrive at the point of observation at the same time. The grey histogram reflects a hypothetical distribution of initiation of hand closure responses. (B) Context in which one size is shown more often than others (the medium size blue ball is more frequent and corresponds to the mean of a Gaussian distribution). If a size prior is assumed then the perceived small and large balls (coded as transparent balls) will be biased towards the most probable one (blue one). The distribution of time action initiation for physically deviant sizes (large-green and small-red balls) will be shifted away from that elicited by the mean (blue). (C) Expected distributions of hand closure initiation according to different optical variables when the the time of action (hand closing) onset is plotted against the ratio size to velocity in log-log coordinates. Different slopes denote the use of different variables (see hypothesis testing for details). (D) Example of an expected distribution of initiation times if prior knowledge is used (e.g. the image is assumed to be caused by the most frequent ball). The correct slope will then be observed (i.e. 1/2 and 1 for 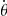 and 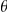 strategies respectively) when the times are plotted against the posterior or perceived size (not the physical ones): the posterior estimate of size 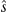 combines prior information (e.g. transparent balls shifted to the mean). Under this assumption, the model that incorporates the prior, besides yielding the correct slopes, predicts less variability (narrower dark grey Gaussian) and larger systematic error for deviant sizes (small-red and large-green balls). If priors are not used at all, accurate slopes are then again expected when the time distribution is plotted against physical size and velocity (like in panel C). True slopes 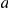 have to be the same when estimated separately on size and velocity (i.e. 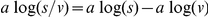 see Eq. 9 for details). (E) Observed distribution of action initiation time as a function of the ratio size to velocity in log-log coordinates for a representative subject.

How do we in ball games know where and when to catch a ball? Answering these questions poses a fundamental problem because the perceptual system has to solve the ambiguity of the retinal image with respect to ball size and distance (see Fig. 1A). Different models can explain how to run close to the landing point of a fly ball [1] on the basis of optical information alone [2], [3] without requiring any metric information of the 3D layout. Once the player is in the right place, precise timing of hand movements with vision is required. Mistaking the true size of the ball would result in failure. How is then a catch timed in spite of this inherent ambiguity? A long-standing solution is the tau-hypothesis [4]–[7]. The 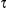 hypothesis holds that time to contact (*ttc*) is computed as the ball's angular size (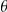) divided by its rate of expansion (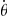). Both angular variables are instantaneously available from the retinal image. For small visual angles and constant speed, 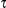 predicts *ttc* independently of ball size and approach velocity. Thus 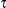 does not require to recover physical information about the ball, even in extended versions of the model [8]. Albeit 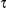 is strictly valid only for small angular sizes and constant approach velocities, studies have provided support for 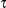 even in cases where acceleration was not negligible [5], [9]. Although 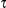 signals *ttc* independently of size and velocity, knowledge of size would be also useful to control grasping movements [7]. However, this aspect has often been neglected in studies that focused on extracting *ttc* from optic flow, (but see [7], [8]). In addition to this problem, 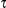 as an all-purpose interception model has been questioned by several studies, suggesting the use of different sources of monocular information [10]–[13]. Furthermore, a binocular correlate of *ttc* was proposed that requires knowledge of physical distance [14]. Although the latter requirement can be interpreted as evidence against 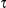, it is possible to use binocular information without the need for recovering distance [15]. The diversity of (mainly monocular) cues we just described is neurophysiologically mirrored by neurons that seem to compute several functions of optical variables in parallel [16]. In addition, the activation of cortical and subcortical areas beyond those involved in visual motion processing [17], [18] reveals a close correspondence between areas that extract *ttc* from expansion with the sensoriomotor systems. In spite of the different alternatives to 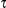 for specifying a timing measure [11], [12], [14], [15], [19], a mechanistic account for the control of action timing has remained elusive. Furthermore a corresponding mechanism would have to deal with image ambiguity while holding size information useful for catching. While 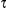-based models predict that there is no ambiguity to solve as to *ttc*, they do not encapsulate useful knowledge about the object to be caught, such as size [20].

Catching, like other natural tasks, implies dealing with variable contexts (e.g. a changing 3D structure) that -once they are projected onto the retina- create ambiguity with respect to the attributes that are relevant for interception (e.g. size, shape, velocity). Coping with such variability is daunting, but sensitivity to the probabilistic structure of relevant objects can help to disambiguate the image [21]. We therefore elaborate on the idea that interceptive timing can benefit from inferring 3D features from the environment in a Bayesian-like way. As a consequence, interceptive behaviour would be consistent with the most likely state of the world that is represented by the (ambiguous) retinal image. By manipulating the probability distribution of ball size, for example, we can verify whether the average behaviour in interception is such that the retinal image was created by the most frequent size (see Fig. 1B). That is, an a priori assumption about the ball size is made on the basis of the experienced distribution. This inference would result in a reduction of response variability at the cost of sometimes failing. For example, larger errors will be committed when a ball deviates (large-green or small-red balls in Fig. 1B) from the prior assumption that underlies the probabilistic structure to which the system is tuned. In this way we extend the use of priors in noisy environments [22], [23] such that they cope with image ambiguity. Whether people would favour this inferential process over 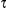 to trade off timing accuracy for reducing variability is unknown.

The simplest mechanism to start a catch is to use a threshold of 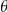, 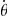 or 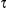. Is a prior assumption (e.g. assuming a given size) compatible with these strategies? 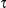, by definition, is impervious to prior knowledge. The use of 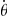, on the contrary, has been reported in the timing of reach onset -but not hand closure- when there was a single ball size [24]. 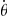 increases temporal precision at the time of action onset, so its use can be promoted by knowledge of size when there is little or minimal time to unfold the action [11]. Nevertheless, the precision when using 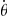 does not depend on the number of different sizes. Interestingly, when using the visual angle 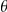 for initiation of catching, then one sees the strongest decrease in the variability of distance at action onset with low size uncertainty. One advantage of using 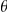 over 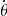 is that its sensory estimate is less noisy. Notice that, with a single ball size, triggering an action when 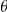 crosses a threshold is equivalent with using a distance threshold [13].

Although it is still under debate which variable eventually initiates an action, various studies highlighted the role of the motor component in interceptive actions. The fact that people move more rapidly when intercepting fast objects [25] suggest an interplay between sensory and motor phases with possible overlapping periods of sensory information acquisition and motor action. The longer one sees a ball's trajectory the lower the error in predicting future positions but always leaving room to perform the action [26]. How the total time is allocated for perception and action reflects knowledge of sensory and movement uncertainty [27].

A single size assumption would then reduce sensory uncertainty, such that a 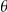 value translates into knowledge of distance, for example. Furthermore, monocular and binocular estimates of approach velocity are possible by knowing size (equation 2 in [11]). This would lead to more robust estimates of *ttc*. Note that when the ball is at a presumably known distance (e.g. because of combining a single size assumption with 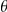), a valid *ttc* estimate only needs approach velocity. Thus 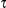 would be redundant. The speed-coupling (e.g. faster movement time for fast balls) reported in previous studies [24], [25], [28] would finally account for timing a catch properly.

In this way, knowledge about size would promote synergies between robust sensory estimates and the kinematics of motor actions. To examine whether this happens, we recorded initiation time and movement time when subjects caught (simulated) balls in a virtual reality setup. Feedback on the accuracy was given. Size and velocity of the balls were drawn from Gaussian distributions with different standard deviations -SD- (large SD implies less reliability). We tested whether subjects initiated hand closure according to 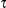, 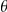 or 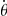 and, if so, whether their responses reflected size or velocity assumptions (see Fig. 1C,D for the predictions). With a model which assumed prior size information, we found that people chose to minimize distance and temporal variability with action initiation and movement time, respectively.

## Methods

### Apparatus and stimuli

Two Barco projectors (iD R600) provided overlaid images for each eye on a back-projection screen (2.44 m width and 1.84 m height) with a resolution of 1024×768 pixels. Each image was refreshed at 85 Hz and circular polarizing filters were used to present stereoscopic stimuli adequate for the user's interpupillary separation and viewing distance (3 m).

A data glove (cyberglove, Immersion) was used to sample finger positions at a rate of 150 Hz. Position data was smoothed using a Butterworth filter (cutoff frequency 10 Hz). We recorded the time of the response initiation or reaction time (elapsed time from the motion onset of the stimulus until the ring finger reached a threshold tangential velocity of 2 cm/s) and closing time that was the elapsed time from the reaction time up to when the ring finger reached a final position consistent with grasping a tennis ball (diameter 66 mm). At the beginning of each session, the glove was calibrated: A comfortable starting open hand position was determined and used to launch the trial. To avoid hand fatigue, subjects could pause the experiment at their will by just keeping the hand comfortably closed at the end of any trial.

Stimuli were created with an OpenGL-based custom program and consisted of textured spheres (like those in Fig. 1A) of different sizes that moved at different constant speeds on a direct collision course (see procedure). Optical expansion therefore was always isotropic and simulated initial time to contact was one of seven possible values (0.64, 0.69, 0.74,0.8, 0.86, 0.93, 0.99 sec). The sphere was presented for random duration according to a flat distribution. This gave random durations between 75% and 85% of the designated initial *ttc* resulting in a range of experimental durations between 0.482 and 0.847 sec (mean of 0.646 sec), respectively. This is preferred to a fixed presentation duration across different initial *ttc* in order to have more equivalent final visual angles and rates of expansion [11].

### Procedure

**Reliability of size and velocity and viewing conditions.** Within a given session, simulated physical sizes and approaching velocities were chosen from different Gaussian distributions with respective mean values and standard deviations. The mean values were 66 mm and 15 m/s for the diameter of the sphere and its approaching velocity respectively. The width of the distribution was chosen according to one of the following conditions. In the size-narrow condition, the diameter was generated from a narrow Gaussian and the velocity from a wider Gaussian, so size was more reliable and velocity less reliable. In the size-wide condition the reliability was reversed: size was drawn from a wider distribution (less reliable) and velocity was drawn from a narrow one (more reliable). The specific deviations depended on individual discrimination thresholds (DT) of size and speed that were obtained by using a Quest [29] procedure in previous sessions. DT were defined as the half difference between 16% and 84% “larger or faster than the standard” responses. Standard deviations of the Gaussians were thus set as the DT multiplied by a factor of 1.2 and 4 for narrow and wide distributions respectively, resulting into about 40% (narrow) and 80% (wide) of the stimuli were perceived as different from the mean.

The two different Gaussian widths were combined with two viewing conditions (binocular and monocular). The disparity of the images was adjusted to match the inter-ocular separation of each subject. In monocular sessions, a null interocular distance was used under the same viewing conditions as in the binocular sessions, and the non-dominant eye was patched in order to avoid conflicts between monocular and binocular cues.

A full control condition was run in which two object sizes (0.058 and 0.074 m) were shown binocularly to help subjects interpret the optic flow. Subjects were explicitly told that they would be shown two different sizes (small and large). The velocity was drawn from the narrow Gaussian with the same mean (15 m/s).

**Task.** Subjects performed interceptive movements with the right hand trying to catch the virtual ball. The hand was placed (but not restricted) in front of the face so that interceptive distance was consistent with the rendered geometry. At the beginning of each trial, the size and velocity of the ball were randomly chosen from their respective distributions. The ball remained still for two seconds in its initial position before it started to move. After this period the ball moved in depth along a direct collision path at the designated velocity and duration. Subjects heard the recorded sound of a real catch if their final position fell in a time window of 30 ms around the actual time to contact. There was a constant delay between the initiation of the closing movement and the output of the sound of 31–35 ms.

For each width of size distribution and viewing condition, respectively, subjects took three consecutive sessions (300 trials each). Ten minute break were allowed between sessions. Sessions with different width size distributions and viewing conditions were run in different days and the order between conditions was randomized across subjects. The first day subjects run a 50 training trials to get used to the task and glove. The order of the control condition sessions (3
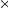
300 trials) were counter-balanced across subjects: presented either at the very beginning or at the end.

**Observers.** Five observers participated in the experiment. All of them were member of the department and had normal or corrected-to-normal vision and were naive with respect to the aims of the experiment. None of the subjects were stereo-blind (StereoFly test, Stereo Optical Co.). Previously, all the subjects gave their informed written consent to participate in the study. The research in this study is part of an ongoing research program that has been approved by the local ethics committee of the University of Barcelona.

### Interception model

From Fig. 1A, the visual angle subtended by, say, the blue ball at time 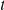 can be approximated in our case by its tangent:
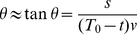
(1)where 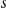 is the diameter of the ball, 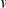 the approaching velocity and 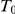 the initial time to contact.

If a visual angle threshold (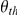) is used to initiate hand-closure movements, the remaining time (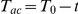) at the time the angle has reached the threshold has a linear relationship with the ratio size to velocity:
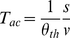
(2)


Taking the time derivative of 2, the rate of expansion 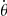 across time would then be approximated by:
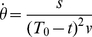
(3)


Then the optical variable rate of expansion 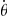 predicts the remaining time (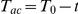) from the moment at which a value 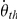 is reached given a known size and velocity:
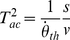
(4)


If we consider a response time delay (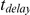) between the time at which 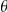 or 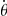 reaches its threshold and the action initiation then we can correspondingly define the time 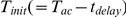 at which the action is initiated relative to the contact time with the eye plane as:
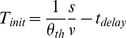
(5)and
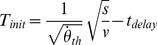
(6)


Therefore if a 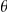 threshold (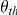) or 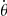 threshold (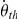) are used to initiate hand-closure movements, the time of action initiation 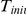 has a linear relationship with the ratio ball size to velocity or the square root of this ratio respectively. Eq. 5 and Eq. 6 both can be more conveniently expressed as a function of 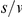 in log-log space:
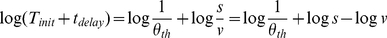
(7)

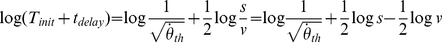
(8)


Both equations can be unified as a function of 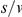 in log-log space:
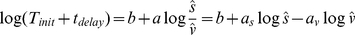
(9)where 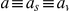 will be 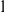 and 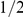 for pure 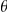 and 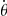 strategies, respectively. Importantly, an accurate estimate of the slopes in the log-log representation should imply that we obtain the same value for 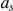 and 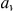 because both parameters denote the same slope 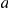 in 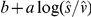. The intercept 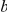 will depend on the threshold values 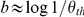 or 
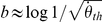
.

Now assume that subjects use *a priori* knowledge of size or velocity. How is this knowledge integrated in Eq. 9? We designate corresponding expectation values as 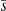 and 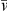, respectively. In other words, 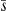 and 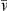 are the mean values of (experimental) distribution of 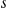 and 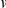, which a subject “learns” during the experiment. We replace thus in the last equation:
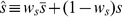
(10a)

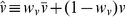
(10b)


### Model fitting procedure

The linear model denoted by Eq. 9 combined with Eq. 10 was used to fit the distribution of initiation times and test the different hypothesis. In Eq. 9, however, 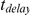 needs to be estimated. In order to do so, we used a procedure similar to that described in [30] to obtain the accurate motor delay from reaction times. In our case we exploit the dependence of the slope 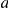 on size and velocity, so that the correct 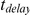 will be that that gives the same slope for size and velocity.

Within nested loops we iterated through values of 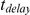, 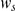 and 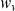. At each such iteration we then fitted the linear model denoted by eq. 9 combined with Eq. 10 to obtain the maximum likelihood estimates of intercept 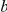 and slopes on size (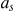) and velocity (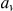). This procedure was carried out separately per each subject, viewing condition and size reliability condition (Fig S1 shows an example). Accurate values of 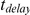 will be those that produce very similar values of the slopes 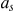 and 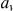 in the fitting procedure. Otherwise, if 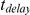 is underestimated then 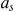 will be overestimated and 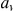 underestimated, or else, if 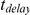 is overestimated then 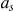 will be underestimated and 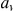 overestimated. Suppose for example that we used a 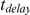 of 50 ms, which is clearly underestimated, then the estimated slope for size 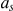 will be larger than the slope for velocity (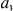).

After an iterative procedure was completed we ended up with a table with different iterated values of 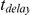, 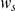, 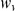, and the corresponding values of size slope 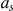 and velocity 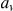 estimated from the fits. We then obtained the crossing values of the slopes (i.e. values where 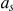 and 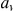 intersected: solid black dots in Fig S1). This subset of slope values resulted in corresponding histograms of slopes, weights (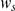 and 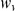) and 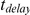 from which we computed the mean values (see inset of Fig S1 for two examples). The mean 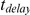 for a given subject and condition was obtained by fitting a log-normal density function to the histogram because it always was positively skewed.

Due to combinatorial explosion, the resolution of the sampling for 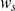 and 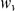 was carried out in an informed way. 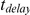 was always sampled from 50 to 300 ms by steps of 5 ms (50 values). However the resolution at which 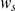 and 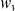 were sampled was lower and incremented when necessary. Initially 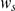 and 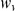 were iterated from 0 to 1 by steps of 0.1 (11 values) to find the regions in 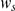 and 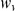 that included intersections between the estimated slopes. Therefore in this initial cycle 6050 (50×11×11) fittings were conducted per combination of subject and conditions. Note that incrementing the resolution to 21 values would result in 22050 fittings. Once a region of interest (with intersections) was detected, we sampled in the appropriate range of the weight parameter that modulated the intersections with a maximum resolution of 0.05. Except for one subject, we never found the same slopes (intersections between 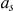 and 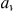) along variations of 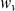.

Eventually we also found spurious intersections (i.e. same values of size and velocity slope that were very unlikely 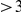). We identified spurious slopes by the concomitant parameters (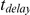 or intercept). They were non-meaningful then.

### Hypothesis testing for optical and prior information

The use of a specific optical variable to start closing the hand can be tested when plotting the time of action initiation plus the response delay 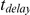 as a function of the ratio size to velocity (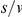) in log-log coordinates. Fig. 1C illustrates the main predictions. A flat distribution (dotted line) is predicted if subjects used 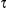 (ratio 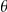 to 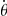). This distribution is independent of variations of size and velocity or the ratio size to velocity. The distribution is expected to have a slope of 1 (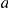 = 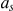 = 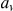 = 1 in Eq. 9) if a pure visual angle threshold initiates hand closure or 1/2 if a rate of expansion threshold is used instead. As these distributions (Fig. 1C) are plotted against the physical values of size and velocity, no prior use of size (or velocity) is taking into account.

Alternatively, subjects could use prior knowledge of size (or velocity) when using an optical variable to start closing the hand. We should then obtain the correct value of the slope when the weight 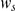 (or 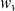) is larger than zero in the fitted model Eq. 9 (Fig. 1D). This implies, as illustrated in Fig. 1D, that the correct slopes are obtained when the distribution of action initiation times are plotted against the perceived (or posterior) values in the abscissa: 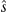 and 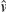 (Eq. 10). This will furthermore result in a better account of the 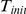 distribution as illustrated by the narrower black Gaussian density in Fig. 1D when prior knowledge is used.

Further validation of the use of a specific optical variable and prior knowledge will come from the interpretation of the intercept in Eq. 9 as this parameter depends on the used threshold value. The parameters estimated as described in the fitting procedure can thus be further tested by comparing their values with the stimulus values at initiation time or with existing literature.

## Results

### Modulation of hand closure initiation by visual angle and prior size

**Figure 2:**
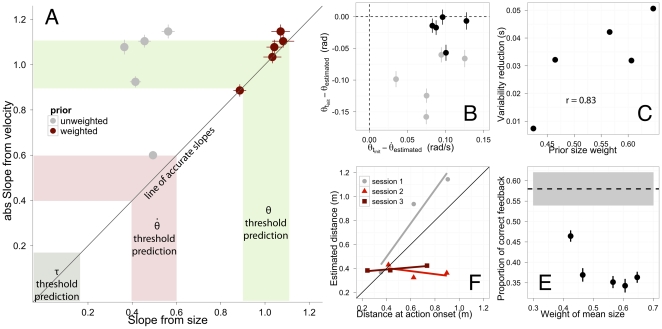
Summary of the results. (A) Slopes (in absolute value) identifying the relationship between time of action initiation and velocity (log-log coordinates) are plotted against the slopes between action initiation and size. Accurate values should be close to the unity line (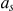 = 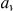 resulting from fitting Eq. 9). Accurate slopes will be close to one if a visual angle threshold is used to start the action of catching (green-shaded area). If a rate of expansion threshold, the slopes will be close to 1/2. Finally the slopes will be close to zero if 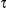 is used. The slopes were obtained either with weighting prior information (red dots) or without (grey dots). (B) Individual differences between actual 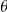 and the estimated 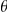 threshold (intercept of Eq. 9) against the same differences for rate of expansion 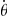. (C) Variability reduction as a function of the weight given to the prior size for each subject. (D) Estimated distance against actual (simulated) distance at action onset. Estimated distance is the slope 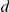 of the linear model that relates velocity and movement time: 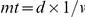. The fit was done separately for different sessions. (E) Proportion of responses that have been initiated within the time window of 30 ms around the actual arrival time as a function of the weight given to the mean size. Single points denote individual subjects and the error bars stand for the binomial 95% confidence intervals. The dashed line is the average proportion of accurate responses in the control condition and the shaded are denotes the 95% confidence limits.

For all subjects the distribution of initiation time showed a linear dependency on variations of target size and velocity suggesting the use of an optical threshold of 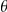 or 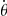 to start closing the hand rather than a 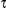-based response. Fig. 1E plots single initiation times against the ratio of physical size and velocity (grey dots) for a representative subject. It is clear from Fig. 1E that action initiation happens later for smaller sizes (or larger velocities). The value of the slope would reveal which optical variable is used. Figure 2A shows a summary of the estimated slopes for all subjects after fitting Eq. 9. The velocity slopes (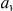 in absolute value) are plotted as a function of the size slopes (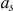). Slopes that had been estimated without considering any prior knowledge (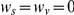) are shown in grey and slopes estimated from fitting 9 with 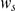 and 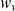 as free parameters are plotted in dark red.

Accurate slopes (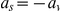) were always obtained when size prior information (not velocity) was weighted in the fit of 9 (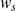 ranging from 0.42 to 0.65). Individual size and velocity slopes were not different from one (t-test against the value of 1, p>0.05), except for subject 3 (slope = 0.88, t(5209) = −2.18, p = 0.03). This slope (0.88) denotes a heavy weighing of visual angle though. Interestingly, this subject yielded the smallest prior size weight (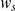 = 0.42) and was the only one to produce a small but significant weight for mean velocity (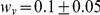). Overall, this pattern is thus very consistent with hand-closure initiation when 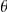 reached a threshold. This trend can also be seen for the subject in Fig. 1E in which initiation times are replotted (red) against their posterior estimates, 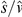 and the slope of the resulting fit is very close to one (reference line has a slope of 1).

In principle, subjects could have estimated physical size more accurately when stereopsis was available. Binocular vision, however, did not change size weighting (repeated measures anova: 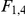 = 0.0921, p = 0.78) or the slope (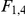 = 0.63, p = 0.47). Neither was the weighting of the size prior affected by the reliability of the size (wide/narrow) distribution (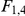 = 4.42 p = 0.103), but size reliability had a marginal effect on slope (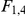 = 6.4051, p = 0.065). These analysis denote a robust effect of a size assumption across different conditions. None of the interactions were significant.

To further validate the slope values (close to one), we can compare the theoretical 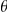 threshold estimated from the intercept of the model Eq. 9 with visual angles (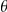) of stimuli at the response time less the estimated time delay. Fig. 2B shows the difference between the actual 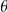 and the estimated 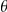 threshold against the same differences observed for rate of expansion (actual less estimated 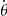) for each subject. As can be seen, the 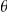 differences are much closer at zero than the 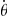 differences when the estimated threshold comes from the model that weights prior size (black dots).

The visual angle strategy gains further support by inspecting the values of the sensori-motor delays 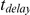 that yielded the same slopes on size and velocity. The estimated individual delays ranged from 0.193 and 0.217 s for all the subjects. As these figures can be interpreted as the time between the moment when the optical information crosses a threshold and the initiation of action, the reported values are consistent with sensorio-motor delays estimated in similar tasks [26].

### Bias and variability

Accurate slopes were not only obtained from considering prior size alone. In addition, resulting models in which size prior was weighted provided a good fit to the data measured by an F-test to individual data (all p<0.05) as well as group data (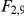 = 85.89, p<0.01)). The model with prior information also outperformed the model in which neither size prior nor velocity prior are weighted. As a consequence, the variability of initiation time distributions was strongly reduced with the model that incorporates prior information (red histogram in Fig. 1E), when compared to the model without prior information. This pattern is consistent across subjects: Fig. 2C shows their individual gains in precision that resulted from weighting the size prior. Individual likelihood ratio tests were conducted in order to rule out the possibility that the reduction of variability in the model that weights prior information is due to the use of two extra degrees of freedom in the fitting procedure (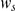 and 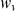). Individual 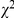 ranged from 7.15 (*p* = 0.028) to 14.48 (*p* 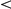
0.001) showing that the performance of the model with prior weights is not merely due to the extra degrees of freedom.

If the pattern shown in Fig. 1E reflects true biased timing responses, systematic temporal errors will accumulate for sizes that deviate from the mean. The estimated delay for the subject in Fig. 1E is 0.210 seconds, so the value of 200 ms in the y-axis denotes an initiation time which is the very close to the actual TTC (10 ms later than contact). As can be observed, late and early responses consistently appear for deviant sizes (small and large size to velocity ratio). Most importantly, we should be able to see reduced accuracy (bigger errors) for subjects that relied more on prior size. Responses initiated within 30 ms around actual contact were provided with auditory feedback. Fig. 2E shows that the proportion of accurate responses was inversely proportional to the weighting of prior size, and it decreased to a level well below the accuracy in the control condition.

One potential confound of the reported pattern comes from the possibility that when subjects responded to a ball size, they identified what they think to be the most and least extreme values and then determine a range (range effect) that serves as a context for judging the ball [31] rather than relying on a perceived size (i.e. by weighting the mean prior distribution). If so, the response to any ball would be a function of relative location within this range, resulting in a simple regression to the mean of the experimental conditions. A range effect would predict different initiation times for similar sizes when they are shown within different contexts (wide versus narrow deviation). We computed the individual means of the initiation time per size bins (four bins), and the difference between narrow and wide deviations was not significant (paired t-test, t = 0.6455, df = 19, *p* = 0.53). Thus a possible range effect can be excluded.

### Closing (movement) time

Reacting to a 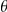 threshold by assuming that a single ball size caused the retinal image strongly reduces distance variability at initiation time. The remaining arrival time at action initiation will then depend on velocity. In order to perform an accurate catch the required time for hand closure should have a positive linear dependency with the reciprocal of the velocity (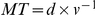) with a slope denoting the distance at initiation time. We performed this linear fit on three different sets of data resulting from binning the physical size into three categories (small, mean and large: means of 5.1, 6.6 and 7.9 cm) for different sessions. The slopes of the fits that denote the estimated distances are the points in Fig. 2D. For session one (red circles) the distances estimated from the movement time closely matched the actual distances at action initiation. These estimates were different for the different size bins. However, for sessions 2 and 3, estimated distances are independent of physical distance at action onset, as indicated by non-zero line slopes (session 2: t = −0.70, p = 0.61; session 3: t = 2.78, p = 0.22). Furthermore line slopes are not different from one another (t = 1.17, p = 0.36). From the estimated distance on the one hand, and visual angle at action onset on the other we can estimate what the assumed size would be. For the session 2, the assumed sizes are 7.7, 5.8 and 6.5 respectively for the small, medium and large size bins. These assumptions further converged to the actual mean (6.6) in the last session: 6.9, 6.9 and 7.6 cm.

### Threshold modulation

**Figure 3:**
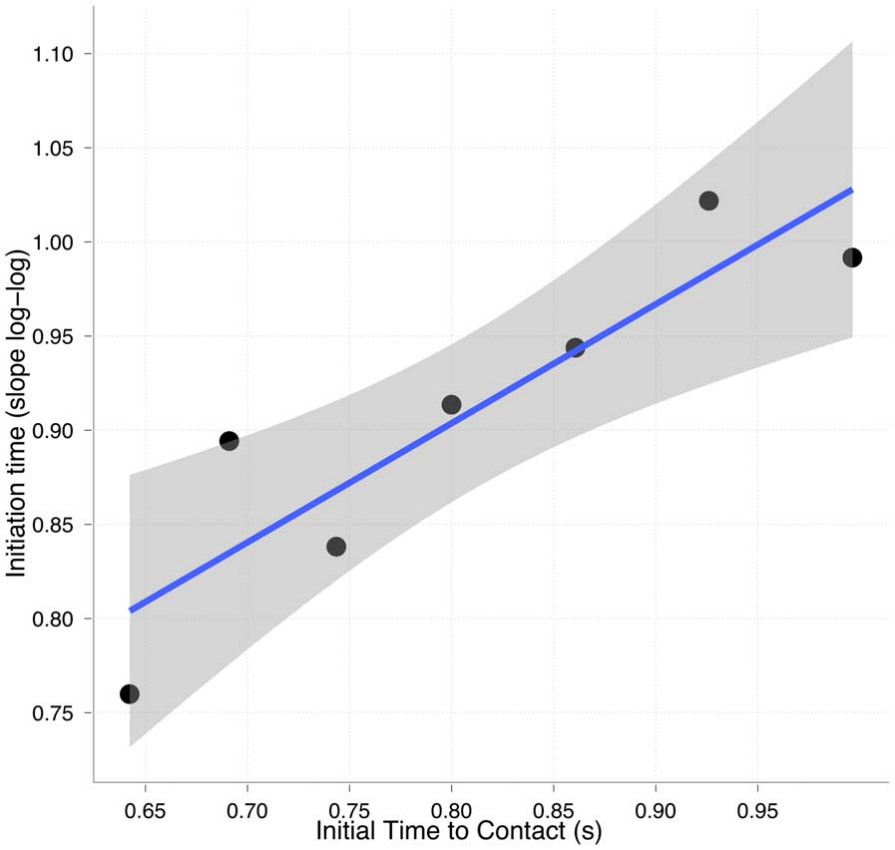
Modulation of the optical variable to initiate the action. The slopes resulting from the fittings of Eq. 9 as a function of the different initial time to contact separately. Data was pooled over subjects.

One can regard the use of this threshold as being too restrictive. If no timing control can be exerted through the motor response (e.g. a button press) the temporal error (task relevant variable) should then be minimised at initiation time as was found in [11] when size was known. This leads to the prediction that people would tend to shift the weight from 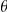 to 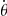 so as to encourage a lower temporal error. We tested this by estimating the slope for different initial time to contacts. As shown in Fig. 3, the slope decreased on average up to 0.75 for the fastest trajectory (0.65 sec of initial time to contact).

## Discussion

Our study shows that -by exploiting size prior information- people can cope with the inherent ambiguity of the retinal image in a systematic way. As a consequence of using prior information, a response bias emerges. This bias leads to an inverse relationship between accuracy and the weight which is given to the prior size. Our results are consistent with previous evidence for using prior information in similar tasks [11], [32], [33], but for the first time it delineates a mechanistic account of the interceptive behaviour that, in addition, keeps relevant 3D information for catching, such as size.

The fact that systematic errors are, in part, caused by favouring a constant size parallels a previous finding in which people consistently neglected (motion and binocular) cues about a scene's 3D structure (motion and binocular cues) in favour of a stable size [34] (when judging an object's size relative to the features of a virtual room). The assumption of a single size can be regarded as subjects holding a strong bias that soon influenced performance, what is consistent with that they learn the mean of a prior distribution very quickly [35]. This influence even persisted in the binocular sessions in which people could potentially estimate physical size more accurately by combining changing disparity and changing optical size [36]. This is not totally surprising as stereopsis is known to have stronger effects on *ttc* judgements when monocular cues are very noisy [14], or when signalling an earlier arrival time than monocular cues [15]. Although we cannot rule out the use of familiar size priors, our results are consistent with subjects pulling actual sizes towards a mean value. A rendered geometry that matches the external world, feedback on successful trials, and combining disparity and visual angle when possible, are sources of information that can help obtain a first anchor of the physical mean size.

One of the benefits of knowing size is that it can be readily transformed into metric information about the reachable space. Such information can be used in addition to modulatory grasping movements. A privileged use of metric information (rather than optical information) has been proposed to perceived the target changing position in interception [37]. Consistent with this idea, it has been shown that judgements of passing distance are encoded in ball size units [8]. In the latter study, real (not simulated) balls were shown.

Acting on a 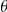 threshold probably emerges because viewing time was long enough to obtain a highly reliable sensory estimate (here distance via a single size assumption) on which initiation could be based, while movement time would reduce the remaining temporal error at action onset. In this sense, our model can be reconciled with previous proposals regarding an optimal combination of the time to glean sensory information, and the time devoted to the motor phase [20], [27]. Action initiation could then be flexibly accommodated (e.g. by varying weights for 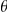 and 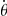 as in [38]) depending on the current temporal constraints. Likewise, our model integrates -within a single account- previous results which have been reported repeatedly like relative size effects, and overestimation of *ttc* for higher speeds (e.g. [10], [11], [38]). The novelty of our approach actually relies on how prior size is embodied in well-known thresholding strategies.

A final question remains as to whether participants respond in a realistic fashion in virtual environments (VE). In [39], it has been reported that action initiation occurred later in the VE than in real catching. Furthermore, catching trajectories could differ quantitatively as well. This latter parameter was here reduced to hand closure, because the hand was already in the point of interest. A later initiation was regarded as being caused by anticipatory mechanisms to the detriment of online control. The reliance of our conclusion with respect to initiation times conforms to size-dependent directional effects that cannot be explained by an overall action initiation delay. The coupling of initiation times and hand closure times as seen in our experiments thus adds plausibility to the ecological relevance of our proposed mechanism.

## Supporting Information

Figure S1
**Size and velocity slopes as a function of different time delays for different weights of prior size (** 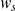 **).** The dashed line denotes velocity slopes (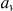 in absolute value to be matched against size slopes) and red solid lines denote size slopes (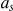) for different 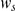 that intersect with velocity slopes. For the sake of illustration only four intersecting curves that correspond to four different values of 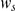 are shown in the figure. In the real fitting the sampling of 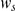 was higher (see text). (insets) histograms of the intersecting size slopes (in red) and the corresponding 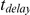 both obtained after a complete iteration procedure ended. Dotted lines in the main figure connect the mean slope on size (estimated from the red histogram) and the corresponding 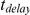 estimated from the blue histogram.(PDF)
