# Supplementary material for: People Favour Imperfect Catching by Assuming a Stable World
Source: PLoS One. 2012 Apr 25;7(4):e35705. doi: 10.1371/journal.pone.0035705 (PMC3338450; doi:10.1371/journal.pone.0035705)
Supplement: Figure S1 — Size and velocity slopes as a function of different time delays for different weights of prior size ( ). The dashed line denotes velocity slopes ( in absolute value to be matched against size slopes) and red solid lines denote size slopes () for different that intersect with velocity slopes. For the sake of illustration only four intersecting curves that correspond to four different values of are shown in the figure. In the real fitting the sampling of was higher (see text). (insets) histograms of the intersecting size slopes (in red) and the corresponding both obtained after a complete iteration procedure ended. Dotted lines in the main figure connect the mean slope on size (estimated from the red histogram) and the corresponding estimated from the blue histogram. (PDF) [file pone.0035705.s001.pdf]

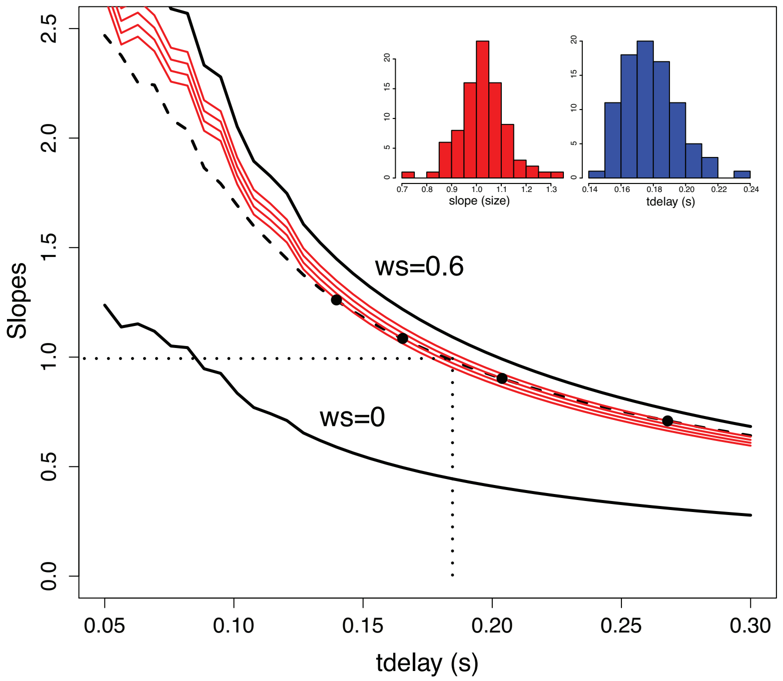

Figure S1: **Size and velocity slopes as a function of different time delays for different weights of prior size (w_s).** The dashed line denotes velocity slopes (a_v in absolute value to be matched against size slopes) and red solid lines denote size slopes (a_s) for different w_s that intersect with velocity slopes. For the sake of illustration only four intersecting curves that correspond to four different values of w_s are shown in the figure. In the real fitting the sampling of w_s was higher (see text). (insets) histograms of the intersecting size slopes (in red) and the corresponding t_{delay} both obtained after a complete iteration procedure ended. Dotted lines in the main figure connect the mean slope on size (estimated from the red histogram) and the corresponding t_{delay} estimated from the blue histogram.
